# Standardized Photographic Views in Oculoplastic Surgery: How to Capture Quality Images Outside a Photographic Studio

**DOI:** 10.7759/cureus.569

**Published:** 2016-04-13

**Authors:** Chin T Ong, George Kalantzis, Jun Fai Yap, Bernard Chang

**Affiliations:** 1 Ophthalmology, University of Malaya; 2 Ophthalmology, St. James University Hospital, Leeds, UK

**Keywords:** clinical photography, digital single lens reflex camera, photodocumentation, standardization

## Abstract

Purpose

The aim of this paper is to demonstrate fundamental photographic techniques and standardized views in oculoplastic disease and surgery outside of a photographic studio.

Methods

A Canon EOS 60D digital single lens reflex (DSLR) camera, which was fitted with a Canon EF-S 60 mm USM macro lens, was used to photograph the subject. A Canon MR-14EX Macro Ringlite was used to illuminate the subject. Informed written consent was obtained for publication of the photographs used in this study. The photographs were taken in an ophthalmology clinic using standardized photographic settings at various magnification ratios. The magnification ratios were recalibrated and adjusted to accommodate the smaller sensor size in-line with standardized medical photography guidelines.

Results

We present a series of core views for various oculoplastic and orbital disease presentations.

Conclusions

It is possible to capture quality standardized digital photographs in a busy clinical environment without the need for a dedicated photographic studio.

## Introduction

Clinical photographs are useful for clinical records, surgical treatment planning, research, audit, education, and in supporting medicolegal cases [1-3]. Standardized clinical photographs are usually taken by medical photographers using professional grade photographic equipment in dedicated photographic studios [1, 4]. However, some clinicians have taken up the role of photographers [1]. Clinicians who personally take patient photographs have an immediate access to photography service and photographs. Since clinicians have a better understanding of the clinical signs to be photographed, they are in a better position to demonstrate the clinical features in front of the camera. In addition, not all hospitals may have access to trained clinical photographers.

We aim to demonstrate fundamental photography techniques and standardized views in oculoplastic and orbital surgery using consumer-grade photographic equipment outside of a photographic studio.

## Materials and methods

A Canon EOS 60D digital single lens reflex (DSLR) camera (Canon, Inc., Tokyo, Japan) was used in this study. This camera has an Advanced Photo System Type C (APS-C)-sized sensor measuring 22.3 mm x 14.9 mm. The camera was fitted with a calibrated Canon EFS-60 mm USM macro lens. A Canon MR-14EX macro ring flash was used to illuminate the subject.

The photographs were taken in an ophthalmology consulting room by one of the authors (CT Ong). A 1 x 1 metre black velvet fabric is used as the background for all of the shots. The fabric is attached to the wall with three foldback clips, which are secured to the wall with reusable adhesive putty. Informed written consent was obtained from the model who posed for the photographs. Informed written consent was also obtained for publication of photos in a journal.

The camera is set to manual exposure mode with an aperture setting of f16 to f22; shutter speed of 1/200th second, auto white balance, RAW file format, evaluative metering, and an ISO of 100. The flash output is determined automatically by the camera via evaluative through the lens (ETTL) metering. The lens is set to manual focus and a sharp focus is achieved by moving the camera forward and backward. The lens has been calibrated by photographing a ruler to achieve a field of view similar to a 35 mm full frame digital camera. The working distance for each reproduction ratio is recorded. The revised reproduction ratio is drawn on the lens barrel. The calibration process is essential to achieve comparable magnification in line with the Westminster reproduction ratio. The Westminster reproduction ratio is used by professional medical photographers, and this standard was based on the 35 mm film camera [5]. The images, which were captured in RAW file format, were downloaded into Adobe Lightroom software version 5 (Adobe Systems, Inc., San Jose, CA) for conversion into TIFF files. All images were converted to grayscale in Adobe Lightroom version 5 software for publishing. No additional digital manipulations were done to enhance the photographs.

## Results

**Figure 1:**
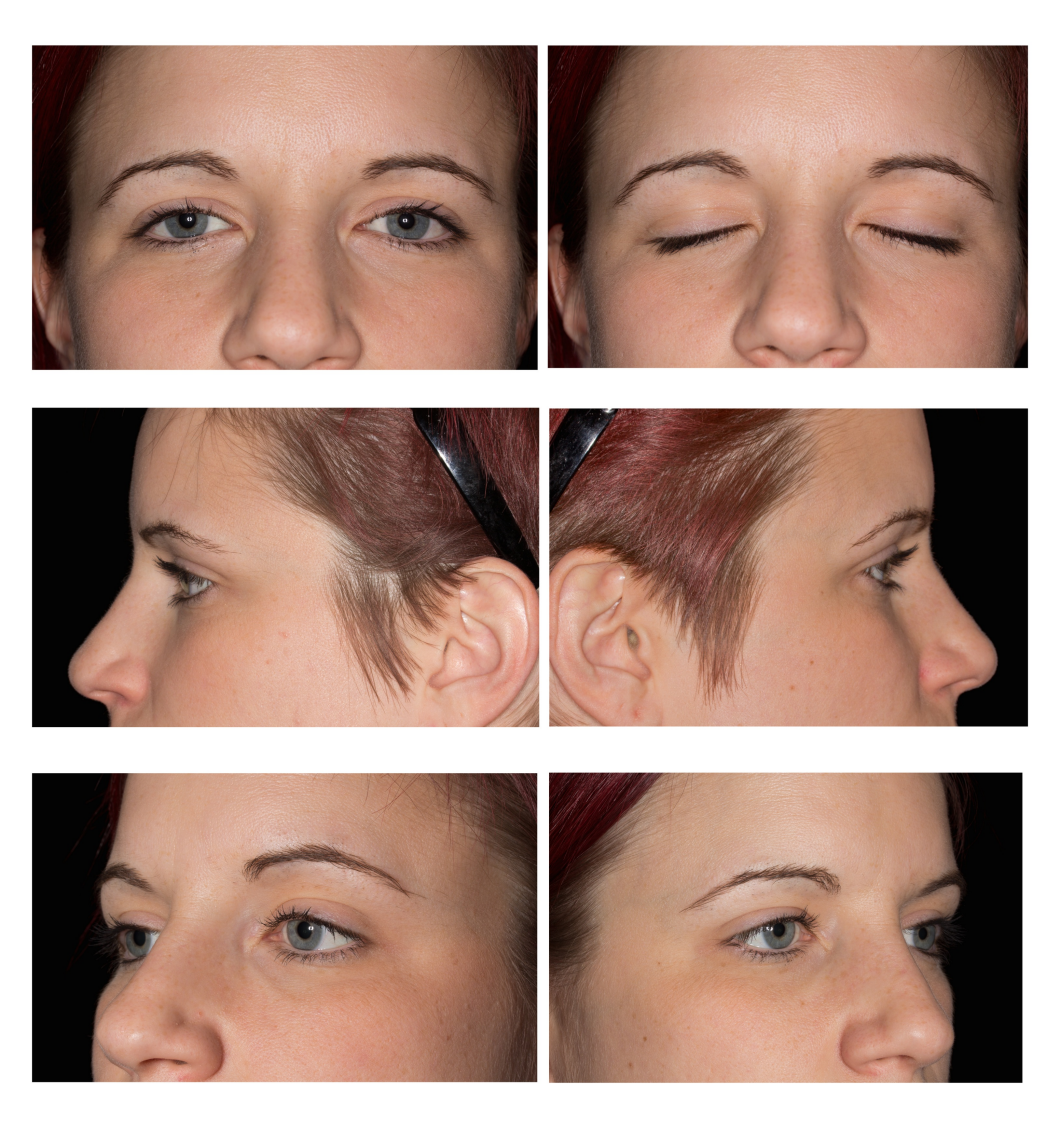
Eyelid views

**Figure 2:**
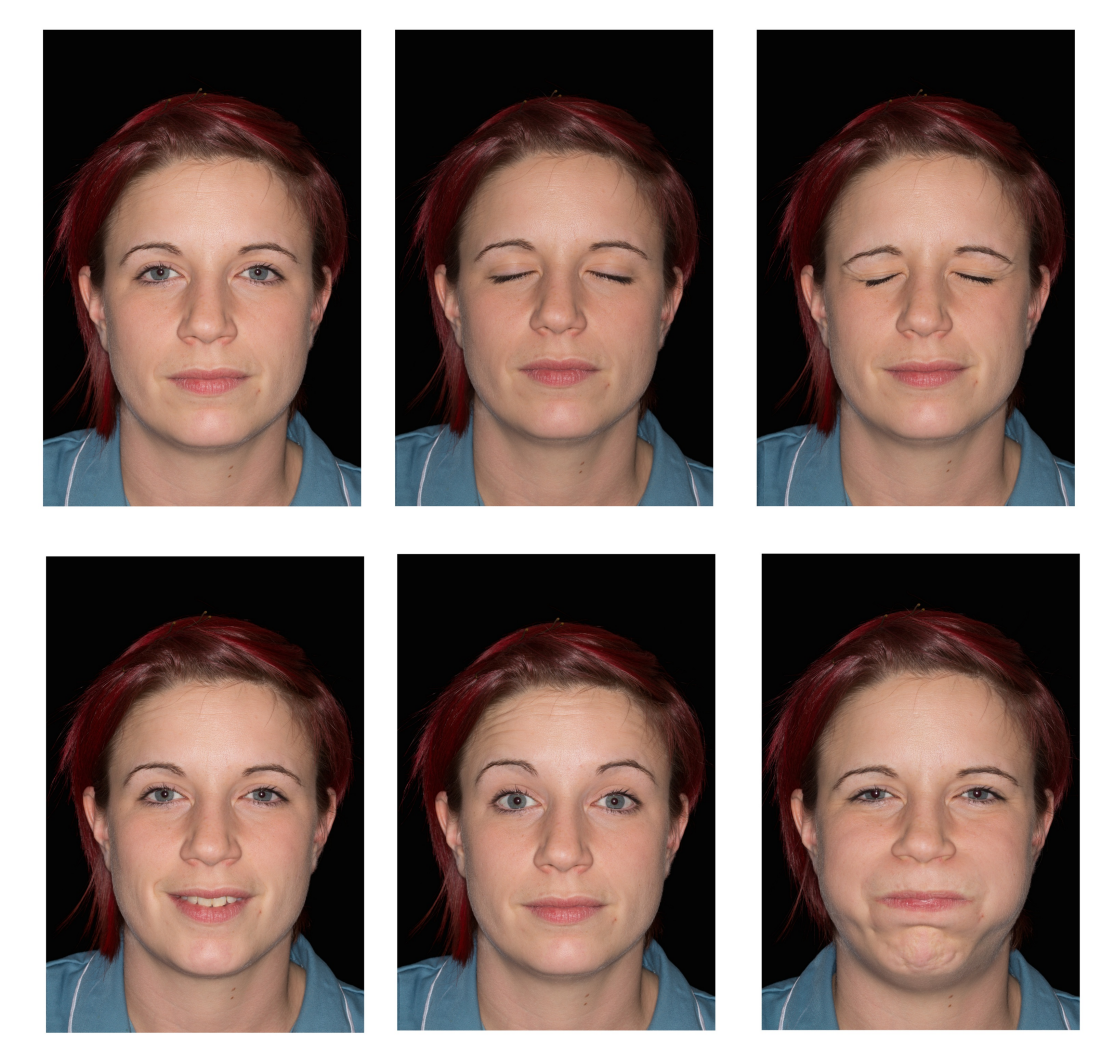
Facial palsy views

**Figure 3:**
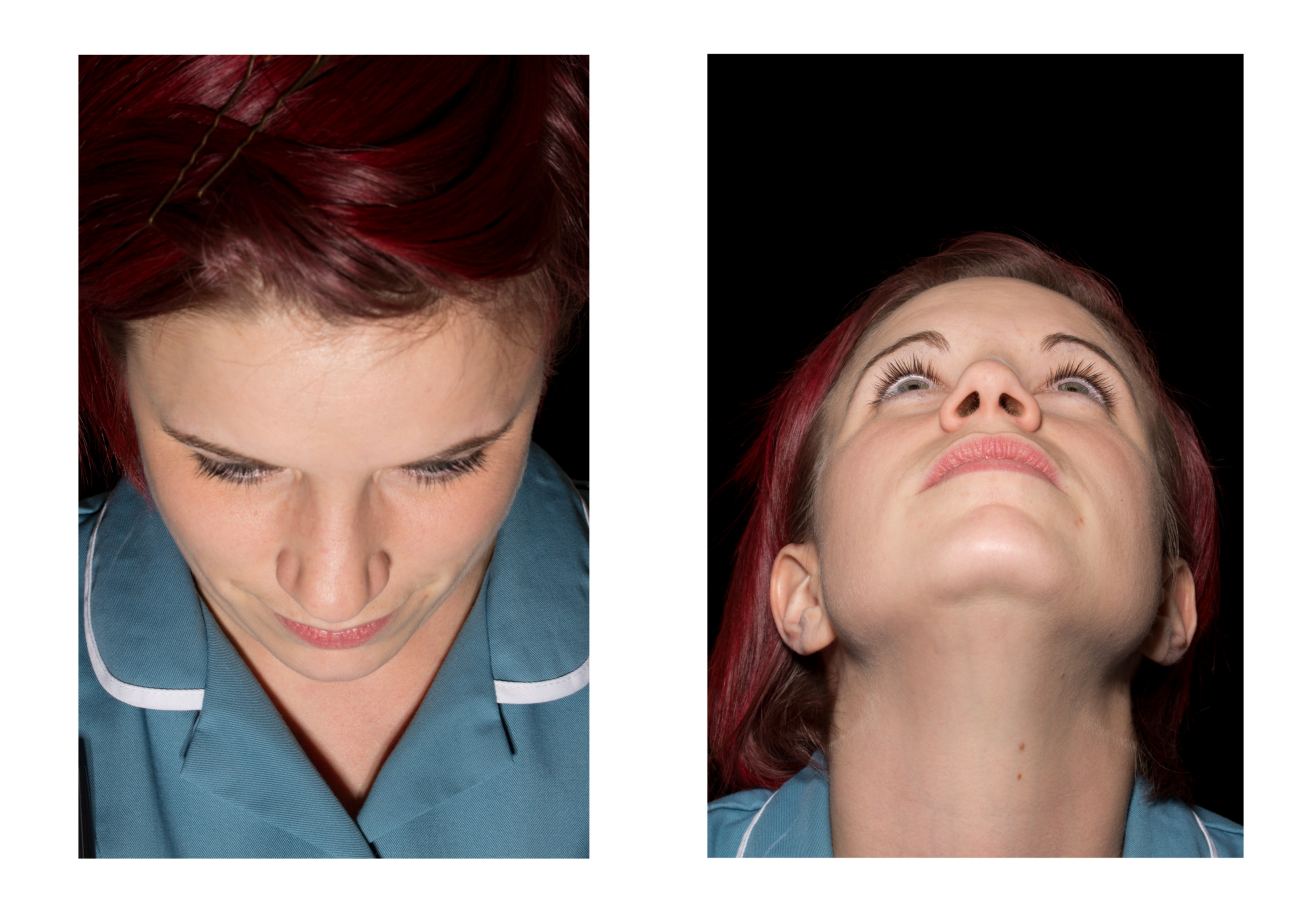
Orbital views

We present a series of core views for various oculoplastic and orbital disease presentations as seen in Figures 1-3.

To obtain a full face view using a 60 mm macro prime lens, the camera needs to be placed 91 cm away from the subject. This will achieve a magnification ratio of 1:8. At this magnification ratio, the camera is able to capture a subject with a dimension up to the 28.8 cm wide and 19.2 cm high. This will enable the whole head and neck to fill the entire frame at portrait orientation. At 1:4 magnification ratio, the camera and subject distance are measured at 50.7 cm. At this magnification, both eyes, eyebrows, and the nose will fill the entire frame. When the magnification is increased further to 1:2, only one eye and the unilateral brow fills the frame. At this magnification, the working distance is measured at 32.3 cm. At the calibrated 1:1 reproduction ratio, the maximum subject dimension, which can be captured on the sensor, is 36 mm x 24 mm. At this level of magnification, only one eye is visible as documented in Table 1.

**Table 1:** Westminster Reproduction Ratio and Working Distance

Westminster Reproduction Ratio	Anatomical Region Photographed	Area of the Subject Photographed	Working Distance (Camera to Subject Distance)
1:1	One eye	36 mm x 24 mm	23.4 cm
1:2	One eye and eyebrow	72 mm x 48 mm	32.3 cm
1:4	Both eyes, eyebrows, and nose	144 mm x 96 mm	50.7 cm
1:8	Full face and neck	288 mm x 192 mm (Portrait mode)	91 cm

## Discussion

Our method allows standardized photographs to be captured quickly in any clinical areas from a busy outpatient clinic to the operating theatre. Clinicians who choose to photograph patients themselves should be registered with the Information Commissioner’s Office as a data controller [6]*.* It is essential to obtain informed consent from patients before taking photographs [7-8].

We use an aperture of f16 to f22 to achieve sufficient depth of field in order to get every detail of the subject in focus. An aperture value of f22 is used at 1:1 reproduction ratio to increase the depth of field. This is to compensate for the reduction of the depth of the field as the camera moves closer to the subject. The camera's built-in electronic through the lens (ETTL) metering does not provide adequate exposure control when dealing with subjects of different skin colours and will have a tendency to overexpose dark skin and underexpose very pale skin [1]. We use a shutter speed of 1/200^th^ second to eliminate the ambient light completely to avoid misleading white balance from ambient light. This means the only light source illuminating the subject is from the camera flash, which has a constant colour temperature of 5500K to 6000K. Since the camera flash has a constant colour temperature, the colour of the photographs can be standardized. We use a low ISO setting as this helps to reduce digital noise on images.

A ring flash is often used in dental photography [9] as the axial light source illuminates oral cavities effectively. The widespread use of this form of flash is often discouraged [10] as it can produce a shadowless image [11], which lacks contrasts definition. However, the author (CT Ong) improves the image contrast by changing the lighting ratios on a ring flash and by moving the flash tubes away from the axis of the lens. When the flash tubes are attached to the front of the lens barrel, the photographer can operate the camera with one hand. The other hand is free to manipulate the patient's eyelids in order to reveal the pathology for the camera. The ability to manipulate the patient’s eyelid is another reason why a 60 mm focal length lens is preferred in oculoplastic photography rather than the more widely used 100 mm macro lens. With a 100 mm lens, the working distance will be too far for the arms to reach the eyelid. As long as the focal length of the lens is at least twice the diagonal of the image sensor in the camera, normal perspective is maintained [12].

A dedicated macro lens is preferred as it allows closer focusing and 1:1 magnification of the object being photographed. Since it is a prime lens with a fixed focal length, it offers superior image quality compared to a zoom lens of similar price range. There is no zoom lens that can achieve a 1:1 macro magnification; therefore, a comparative study on the two lenses is not possible. The Canon EFS-60 mm USM macro lens used in this study has been calibrated to achieve a field of view similar to a 35 mm full frame camera. A full frame camera is usually more expensive as it has a larger image sensor (35 mm x 24 mm). A larger sensor means the camera can capture a wider field of view compared to a camera with a smaller sensor using the same lens. In our study, we used an APS-C sensor camera with a smaller sensor size measuring 22.3 mm x 14.9 mm. The Westminster Scale of Reproduction has been the industry standard for many years and is based on the 35 mm format. The linear scale etched on the Canon EF-S 60 mm macro lens mounted on an APS-C sensor camera does not match the Westminster Scale on a full frame (35 mm format) camera. Therefore, the lens has to be calibrated to produce an alternative linear scale, which provides a field of view equivalent to a full frame camera. The details of the calibration technique have been published [5, 13].

The ETTL pre-flash may trigger the blink reflex leading to an inaccurate documentation of patients with ptosis or lid retraction. This limitation can be overcome by setting the flash output manually or by pressing the flash exposure lock button before pressing the shutter release button. This separates the pre-flash from the main flash to minimise capturing the patient’s blink on camera. The author (CT Ong) prefers to frame the shot via the optical viewfinder. An optional focus screen with grids has been installed in the camera to facilitate accurate framing of the shot [1-2]. These grids are often used by landscape and architectural photographers to align the horizon. The grids are essential in keeping the Frankfurt plane (auriculo-orbital plane) horizontal. This plane is regarded as the anatomical position of the human skull [14]. It runs from the upper border of the tragus to the ipsilateral inferior orbital rim. In patients with no sign of strabismus, the corneal light reflexes on each eye can be used as horizontal reference points. Eyebrows, nose, ears, and canthi are highly variable and often asymmetrical anatomical landmarks, which should be used with caution when framing a shot.

RAW file format contains all the data captured by the image sensor while a JPEG file is a compressed image file to conserve disk space. A RAW file can be converted to any file format, but not vice versa. A RAW file contains all the colour information of the image, and the desired white balance can be applied or adjusted on a computer following image capture. Adobe Lightroom is a RAW image processing software that maintains a database of all the images captured. We advocate importing images from memory disks into this software and tagging the images with keywords to facilitate image retrieval at a later date. The image files should be backed up and encrypted in line with data protection and information governance regulations.

## Conclusions

The key to medical photography is standardization. It is possible for clinicians to obtain quality clinical photographs without a dedicated studio if the recommended general medical photography principles are followed.

## References

[1] Archibald DJ, Carlson ML, Friedman O (2010). Pitfalls of nonstandardized photography. Facial Plast Surg Clin North Am.

[2] Becker DG, Tardy ME Jr (1999). Standardized photography in facial plastic surgery: pearls and pitfalls. Facial Plast Surg.

[3] Ellenbogen R, Jankauskas S, Collini FJ (1990). Achieving standardized photographs in aesthetic surgery. Plast Reconstr Surg.

[4] Morello DC, Converse JM, Allen D (1977). Making uniform photographic records in plastic surgery. Plast Reconstr Surg.

[5] Young S, Lake A (2008). Calibrating lenses for standard scales of reproduction with digital SLR cameras. J Vis Commun Med.

[6] Commissioner's Commissioner's (2015). The Data Protection Act 1998. Internet.

[7] 1 1 (2015). Making and using visual and audio recordings of patients. Internet.

[8] Institute Institute (2015). IMI National Guidelines Consent to Clinical Photography. Clinical Photography. 2006.

[9] Fan PP (1998). Choosing the right clinical camera. Part I. Oral Health.

[10] Institute Institute (2015). IMI National Guidelines
Ophthalmic Imaging. Imaging.

[11] Vachiramon A, Wang WC, Tovee M (2006). A lighting approach for clinical photographs of the face. J Contemp Dent Pract.

[12] Nayler JR (2003). Clinical photography: a guide for the clinician. J Postgrad Med.

[13] Young S (2001). Maintaining standard scales of reproduction in patient photography using digital cameras. J Audiov Media Med.

[14] Schaaf H, Streckbein P, Ettorre G, Lowry JC, Mommaerts MY, Howaldt HP (2006). Standards for digital photography in cranio-maxillo-facial surgery--part II: additional picture sets and avoiding common mistakes. J Craniomaxillofac Surg.

